# What Is “Being Successful”?

**Published:** 1917-02

**Authors:** J. F. Canover

**Affiliations:** Calman, Iowa


### WHAT IS "BEING SUCCESSFUL"?

BY J. F. CANOVER, D.D.S., CALMAN, IOWA.

Success! that's the thing we all are seeking, but sometimes we do not know her when we meet her. Oft-times we salute a stranger. I am not sure that I know the lady, but I have a notion that I know what she looks like. Some have said that success is the attainment of an ideal; others, that it is the amassing of a fortune; or the achievement of fame; or the doing of what one sets out to do. Suppose you get started in the wrong direction; perseverance might land you in the pen; I have a notion that it is none of these. True success brings contentment. Does the attainment of an ideal bring contentment? That depends entirely on the ideal. Does money bring contentment? Watch the men who have it. They work like slaves to get it, still harder to keep it, and can't sleep nights for fear they will lose it. How about fame? The emptiest bubble ever blown. Ask Napoleon at St. Helena. Ask Benedict Arnold as they wrapped the American flag about his poor wasted body. And it might not be amiss to interrogate Teddy.

What is it then that we must possess to be successful? Again I do not know, but I have another notion. It seems to me that the basis of success is health, using that word in its broadest sense. Health may be divided into three departments, if you please. First, moral health, which manifests itself in unselfish service to mankind. Second, mental health, which manifests itself in the discovering of materials and methods necessary to this service, and third, physical health, which manifests itself in untiring energy for the performance of service. The discussion of these three states of health must be very brief (for the sake of your health).

As previously stated, morality is conformity to conscience. Moral health then is the result of a good conscience, a true conception of the essentials of man's relation to man. That this condition may obtain, the individual must be well born, a debt the world owes to every child coming into it, but one which in so many cases is never paid. He must be given a prior environment—a good home. Every child is entitled to a good home, yet how many there are who never know the meaning of the term. He must have a common interest with his fellows. That is friendship. Robert Louis Stevenson said that no man is worthless who has a friend. Will either fame or money get friends? Verily, NO. The only way to have friends is to be a friend. Be a friend to the whole wide world; give to it the best service at your command; live so that you will leave the world better than you found it. That is true success and the striving for it will bring contentment.

The individual must receive an adequate education. This takes us into the realm of mental health which is ministered to by our schools, general and special. Nothing need be said to emphasize the necessity of intelligence relating to any and all of life's activities. Lastly, we come to physical health. No matter how complete the individual's mental and moral equipments may be if he has not the physical ability to apply them in the form of activity he will accomplish but little.

Now, to bring this subject to a more intimate and personal relationship. Did it ever occur to you that to care for your own physical welfare is as much a part of your professional work as is the doing of operative or prosthetic work? You can best conserve the health of others by first conserving your own. The basis of human existence is food. I used to think that I knew something about the subject of proper food, but a cantankerous stomach has to a great degree dispelled that illusion.

Again I have a few notions. We often hear of good boys and bad boys, but all boys are good if given a chance, the so-called bad boy being a good one with proper surroundings. So it is with foods. All foods are good, but they go wrong when they get in bad company. Instead of foods not agreeing with us, as we say, the fact is they do not agree with each other. Foods are merely chemicals, and you know what the results are of combining incompatibles. In like manner, when we throw a lot of unfriendly foods together in our stomachs they quarrel and throw the furniture at each other. The eating of foods that are harmonious chemically produces harmony in the digestive tract and harmony is health. Back to nature is a popular health slogan of today. Just how much virtue there is in this stuff I do not know. Indeed, is there any such thing as getting back to nature? Isn't it getting ahead to nature? Are we sub-natural or super-natural? One thing I think I do know, and that is that fresh air, pure water, shimmering sunshine, and harmonious sights and sounds are necessary to good health. Not physical health only, but to mental and moral as well. And I further know that these blessings are to be found in nature's great out of doors.—*Dental Digest.*

